# A New GNSS Interference Detection Method Based on Rearranged Wavelet–Hough Transform

**DOI:** 10.3390/s21051714

**Published:** 2021-03-02

**Authors:** Kewen Sun, Tengteng Zhang

**Affiliations:** 1 School of Computer and Information, Hefei University of Technology, Tunxi Road 193, Hefei 230009, China; 2019170914@mail.hfut.edu.cn; 2 Intelligent Interconnected Systems Laboratory of Anhui Province, Hefei University of Technology, Tunxi Road 193, Hefei 230009, China; 3 State Key Laboratory of Satellite Navigation System and Equipment Technology, Zhongshan West Road 589, Shijiazhuang 050002, China

**Keywords:** global navigation satellite system (GNSS), interference detection, rearrangement, wavelet transform, rearranged wavelet–Hough transform (RWHT)

## Abstract

Since radio frequency interference (RFI) seriously degrades the performance of a global navigation satellite system (GNSS) receiver, interference detection becomes very important for GNSS receivers. In this paper, a novel rearranged wavelet–Hough transform (RWHT) method is proposed in GNSS interference detection, which is obtained by the combination of rearranged wavelet transform and Hough transform (HT). The proposed RWHT method is tested for detecting sweep interference and continuous wave (CW) interference, the major types of GNSS interfering signals generated by a GNSS jammer in a controlled test bench experiment. The performance of the proposed RWHT method is compared with the conventional techniques such as Wigner–Ville distribution (WVD) and Wigner–Hough transform (WHT). The analysis results show that the proposed RWHT method reduces the influence of cross-item problem and improves the energy aggregation property in GNSS interference detection. When compared with the WHT approach, this proposed RWHT method presents about 90.3% and 30.8% performance improvement in the initial frequency and chirp rate estimation of the GNSS sweep interfering signal, respectively. These results can be further considered to be the proof of the validity and effectiveness of the developed GNSS interference detection method using RWHT.

## 1. Introduction

With the increasing global navigation satellite system (GNSS) applications in various civil and military fields, reliable navigation and positioning performance has become a key and important requirement for GNSS receivers [1,2,3,4]. The direct sequence spread spectrum (DSSS) scheme which has been used in most of the satellite navigation systems spreads the received GNSS signal power over a wider bandwidth; this ensures a dispreading gain in the GNSS receiver, which can reduce impairments caused by the undesired disturbing signals [1]. Although GNSS has a certain capability to be immune from interference, due to the reception of the very low GNSS signal power, intentional or unintentional radio frequency interference (RFI) can cause serious performance degradation of a GNSS receiver; for instances, RFI may cause degradation of GNSS signal quality, serious errors in navigation and timing results, and even completely loss lock of the receiver [2,3,4,5,6]. Therefore, interference detection has become a critically important role for GNSS applications [7,8,9,10,11,12,13,14,15,16].

The commonly used methods in GNSS interference detection mainly include automatic gain control (AGC) method, time domain methods, and frequency domain methods. AGC adjusts the GNSS input signal level to the range of the analog-to-digital converter (ADC), which performs interference detection by monitoring the AGC level [17,18,19]; however, in a weak interference environment, the detection performance of the AGC method is significantly reduced. For the time domain method, this method can be implemented at the digital intermediate frequency (IF) level after the ADC at the front end of the GNSS receiver; this time domain method is only effective for narrowband RFI sources, because broadband interference cannot be easily distinguished from thermal noise [20,21]. For the frequency domain method, due to its spectral characteristics, it is usually used to detect narrowband carrier interference [22,23,24]. Concerning GNSS interference detection by using time domain methods or frequency domain methods, they cannot fully describe the nature and characteristics of the time-varying interference present in the received GNSS signals [13].

Presently, the research topic on time-frequency (TF) transforms in GNSS interference detection has obtained increasing attention [4,7,8,9,10,11,12,13]. For example, the spectrogram and Wigner–Ville distribution (WVD) were used for interference detection in GNSS receivers, due to the limitation of Heisenberg’s uncertainty principle, the spectrogram method has a TF resolution trade-off problem, thereby providing poor TF localization characteristics; although WVD presents good time-frequency aggregation property, in the case of multi-component signals, serious cross-interference terms occur in the TF plane [7,10,11,12,13,14,15,16].

To deal with the TF resolution trade-off problem with the spectrogram and the cross-term problem with the WVD, the Wigner–Hough transform (WHT) has been adopted [25,26,27]. In the detection of linearly frequency modulated (LFM) interfering signal for GNSS applications, Hough transform converts the problem of global straight-line detection in the image space to the local peak detection problem in the parameter space [15,16]. Although WHT shows improved performance in interference detection [15], under low signal-to-noise ratio (SNR) scenario, false WHT peak occurs in the parameter space due to strong noise, resulting in interference detection error.

The rearrangement operation can be combined with the TF representations for signal detection applications [8,9,28,29]. To improve GNSS interference detection performance, in this paper, the rearrangement operation has been introduced to the continuous Wavelet transform (CWT), obtaining the rearranged wavelet transform, which is beneficial for the significant improvement of the TF aggregation property in the TF plane; then, the obtained rearranged wavelet transform has been further combined with the Hough transform, which is very effective to deal with the cross-term problem; in this way, a new rearranged wavelet–Hough transform (RWHT) method has been proposed in interference detection for GNSS receivers particularly working in challenging interfering environments, which can be considered to be the main novelty of this paper.

In this paper, first, the cross-term problem with the conventional TF analyses such as WVD used in GNSS interference detection has been discussed and then the Hough transform has been adopted to overcome this tough cross-term problem; second, the rearranged wavelet transform has been developed to improve the TF resolution in the TF plane; finally, the rearranged wavelet transform has been further combined with the Hough transform obtaining the novel RWHT which is expected to improve the interference detection performance for GNSS receivers. 

To prove the validity and effectiveness of the proposed RWHT-based GNSS interference detection method, the interference detection experiment has been performed by using the real GPS L1-C/A signal collected in the presence of sweep interference or continuous wave (CW) interference. The analysis results have shown that the proposed RWHT technique effectively suppress the cross-terms in the bilinear TF distributions and greatly enhances the TF localization property in the TF plane, which significantly improves the interference detection performance for GNSS receivers particularly working in the difficult jamming scenarios.

This paper is organized as follows: Section 2 introduces the models and methods used in GNSS interference detection; Section 3 analyzes the GNSS interference detection results; the discussion has been made in Section 4; finally, the conclusion has been addressed in Section 5.

## 2. Models and Methods

### 2.1. GNSS Signal and Interference Model

In an interfering environment, the model of the signal at the input of a GNSS receiver can be represented as [10,11,12,13,15]:

(1) $$y_{RF}\left(t\right)=\overset{N_{S}}{\underset{i=1}{\sum }}r_{RF,i}\left(t\right)+\eta_{RF}\left(t\right)$$

where $r_{RF,i}\left(t\right)$ represents the *i*th GNSS signal ($i=1,\;2,\;\cdots ,\;N_{s}$), $N_{s}$ denotes the number of satellites in view, and $\eta_{RF}\left(t\right)$ is the disturbing term. 

When a single useful signal is considered, the GNSS signal transmitted by the *i*th satellite can be given as [10,11,12,13,15]:

(2) $$r_{RF,i}\left(t\right)=A_{i}c_{i}\left(t-\tau_{i}\right)d_{i}\left(t-\tau_{i}\right)\cos\left[2\pi \left(f_{RF}+f_{d,i}\right)t+\varphi_{RF,i}\right]$$

where:

- $A_{i}$ is the amplitude of the *i*th useful GNSS satellite signal;
- $\tau_{i}$ is the propagation delay for the *i*th satellite signal;
- $c_{i}\left(t-\tau_{i}\right)$ denotes the Pseudo Random Noise (PRN) code sequence extracted from a family of quasi-orthogonal codes modulated by adopting rectangular pulses. For the simple case of the GPS L1 signal, $c_{i}\left(t\right)$ represents the C/A code;
- $d_{i}\left(t\right)$ represents the navigation data message;
- $f_{RF}$ denotes the carrier center frequency of the GNSS signal. When considering GPS L1 signal, $f_{RF}=f_{L1}=$ 1575.42 MHz;
- $f_{d,i}$ denotes the Doppler frequency shift affecting the ith useful GNSS signal;
- $\varphi_{RF,i}$ is the initial carrier phase offset for the *i*th useful GNSS signal.

In general, the disturbing term $r_{RF}\left(t\right)$ can be expressed as [10,11,12,13,15]:

(3) $$\eta_{RF}\left(t\right)=j_{RF}\left(t\right)+w_{RF}\left(t\right)$$

where $j_{RF}\left(t\right)$ is the non-stationary interfering signal and $w_{RF}\left(t\right)$ is the GNSS receiver thermal noise which is usually in the form of a zero-mean stationary Additive White Gaussian Noise (AWGN).

Potentially, there are different forms of interfering signals generated by RFI sources. In this paper, without loss of generality, the interference term $j_{RF}\left(t\right)$ can be considered to be a sweep interference (linear chirp), usually frequency modulated with near constant amplitude.

Sweep interference, i.e., LFM interference, is considered to be one of the main types of interfering signals. When considering GNSS applications, sweep interference is regarded as an interference pattern that performs periodic linear scanning of the GNSS target frequency band, thereby effectively reducing the reliability or safety of satellite navigation and positioning services in the target frequency band. Sweep interference can be expressed by sinusoids in the time domain as:

(4) $$j_{RF}\left(t\right)=A_{inst}\left(t\right)\cos\left[2\pi \int_{0}^{t}f_{inst}\left(t\right)dt+\varphi_{0}\right]$$

where $A_{inst}\left(t\right)$ is the carrier amplitude of the sweep interfering signal, $f_{inst}\left(t\right)$ is the instantaneous frequency of the sweep interference, and $\varphi_{0}$ denotes the initial carrier phase of the sweep interference, which can be considered to be a random variable presenting a uniform distribution in the range $\left[-\pi ,+\pi \right)$.

The instantaneous frequency $f_{inst}\left(t\right)$ of the linear chirp can be expressed as:

(5) $$f_{inst}\left(t\right)=f_{0}+kt\;0\leq t\leq t_{j}$$

where $f_{0}$ is the initial frequency, $t_{j}$ denotes the frequency sweep period for the interfering signal, and k represents the chirp rate or the frequency modulation rate.

The CW interference can be considered to be a special case of the sweep interference with a fixed carrier frequency $f_{0}$; in this case, $j_{RF}\left(t\right)$ can be expressed as [13]:

(6) $$j_{RF}\left(t\right)=A_{CW}\left(t\right)\cos\left[2\pi f_{CW}\left(t\right)t+\varphi_{0}\right]$$

where $A_{CW}\left(t\right)$ is the amplitude of the CW interfering signal, $f_{CW}\left(t\right)$ denotes the center frequency of the carrier, and $\varphi_{0}$ represents the initial carrier phase of the CW interference.

The input signal $y_{RF}\left(t\right)$ defined in Equation (1) is filtered and down-converted by the GNSS receiver front end. Then, the received GNSS signal before the ADC is expressed as [10,11,12,13,15]:

(7) $$y\left(t\right)=\sum_{i=1}^{N_{s}}r_{i}\left(t\right)+\eta \left(t\right)=\sum_{i=1}^{N_{s}}A_{i}{\tilde{c}}_{i}\left(t-\tau_{i}\right)d_{i}\left(t-\tau_{i}\right)\cos\left[2\pi \left(f_{IF}+f_{d,i}\right)+\varphi_{i}\right]+\eta \left(t\right)$$

where $f_{IF}$ is the intermediate frequency (IF) of the GNSS receiver; ${\tilde{c}}_{i}\left(t-\tau_{i}\right)$ represents the spreading code sequence after filtering in the GNSS receiver front end. Here the effect of the GNSS receiver front-end filter is neglected assuming the simplifying condition ${\tilde{c}}_{i}\left(t\right)\approx c_{i}\left(t\right)$; and $\eta \left(t\right)$ represents the disturbing component after down-conversion and filtering, $\eta \left(t\right)=j\left(t\right)+w\left(t\right)$.

To avoid the cross-terms caused by the interaction between the positive and negative frequency parts of the spectrum, an analytic form of the received GNSS signal has been proposed [7,10,11,12,13,15], expressed as:

(8) $$y_{a}\left(t\right)=y\left(t\right)+j\hat{y}\left(t\right)$$

where j is the imaginary root unit and the analytic signal $y_{a}\left(t\right)$ contains a real part $y\left(t\right)$ representing the original GNSS signal and an imaginary part $\hat{y}\left(t\right)$ denoting the Hilbert transform of $y\left(t\right)$.

### 2.2. Time Frequency Analyses

#### 2.2.1. Wigner–Ville Distribution (WVD)

WVD is a commonly used TF distribution for analyzing non-stationary time-varying signals, which belongs to a typical quadratic or bilinear TF representation since the analyzed signal is used twice in the calculation. The WVD can be defined as the Fourier transform of the time-dependent instantaneous auto-correlation function $R_{y}\left(t,\tau \right)$ of the analyzed signal, given as:

(9) $$WVD\left(t,\omega \right)=\int_{-\infty }^{+\infty }R_{y}\left(t,\tau \right)e^{-j\omega \tau }d\tau =\int_{-\infty }^{+\infty }y_{a}\left(t+\frac{\tau }{2}\right)y_{a}^{*}\left(t-\frac{\tau }{2}\right)e^{-j\omega \tau }d\tau$$

where the instantaneous correlation function $R_{y}\left(t,\tau \right)$ is equal to $y_{a}\left(t+\frac{\tau }{2}\right)y_{a}^{*}\left(t-\frac{\tau }{2}\right)$, $y_{a}\left(t\right)$ represents the analytic signal, τ is called the lag variable.

Although WVD has many nice properties and provides nearly the best TF resolution among all the TF analysis techniques, its main drawback comes from cross-term problem due to its bilinearity nature [30,31]. Consider the signal $y\left(t\right)=y_{1}\left(t\right)+y_{2}\left(t\right)$, where $y\left(t\right)$, $y_{1}\left(t\right)$ and $y_{2}\left(t\right)$ are analytic. Expanding the instantaneous auto-correlation function of $y\left(t\right)$, we can obtain

(10) $$R_{y}\left(t,\tau \right)=R_{y1}\left(t,\tau \right)+R_{y2}\left(t,\tau \right)+R_{y1y2}\left(t,\tau \right)+R_{y2y1}\left(t,\tau \right)$$

where $R_{y1y2}\left(t,\tau \right)$ and $R_{y2y1}\left(t,\tau \right)$ are respectively the instantaneous cross-correlation functions (e.g., $R_{y1y2}\left(t,\tau \right)=y_{1}\left(t+\tau /2\right)\;y_{2}^{*}\left(t-\tau /2\right)$). 

Taking Fourier transforms of Equation (9) with respect to τ, we can have

(11) $$WVD_{y}\left(t,\omega \right)=WVD_{y1}\left(t,\omega \right)+WVD_{y2}\left(t,\omega \right)+2Re\left\{WVD_{y1y2}\left(t,\omega \right)\right\}$$

where $WVD_{y1}\left(t,\omega \right)$ and $WVD_{y2}\left(t,\omega \right)$ are the WVDs of $y_{1}\left(t\right)$ and $y_{2}\left(t\right)$, respectively, and the last term is considered to be the cross-WVD (XWVD) between $y_{1}\left(t\right)$ and $y_{2}\left(t\right)$, provided as:

(12) $$WVD_{y1y2}\left(t,\omega \right)=\int_{-\infty }^{+\infty }y_{1}\left(t+\frac{\tau }{2}\right)y_{2}^{*}\left(t-\frac{\tau }{2}\right)e^{-j\omega \tau }d\tau$$

From Equation (11), it is easy to know that the WVD of the sum of two signals is not only the sum of their corresponding WVDs, but also of their XWVD. This means that the spectrum energy density of the sum of two signals does not reduce to the sum of the individual densities (unless the signals are spectrally disjoint). If $y_{1}\left(t\right)$ and $y_{2}\left(t\right)$ are mono-component signals, $WVD_{y1}\left(t,\omega \right)$ and $WVD_{y2}\left(t,\omega \right)$ are the auto-terms, while 2ReWVDy1y2t,ω is called the cross-term.

**Figure 1:**
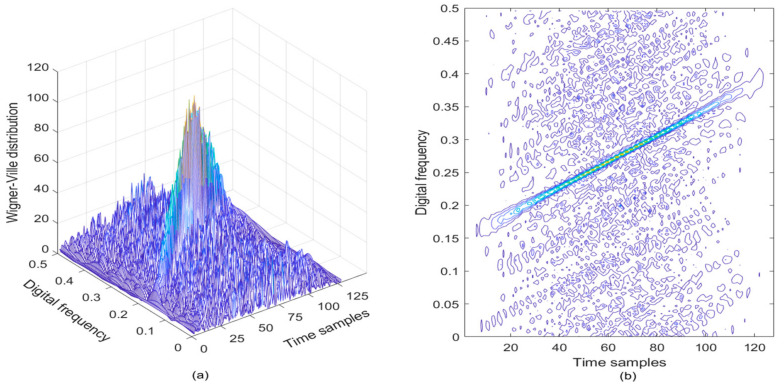
The WVD of LFM signal in the presence of AWGN, where the SNR is set to be 3 dB. (**a**) WVD of the noisy LFM signal; (**b**) Contour of WVD.

As a result, if a signal contains more than one component in the TF plane, its WVD suffers from spurious features containing cross-terms that occur halfway between each pair of auto-terms. As an example, the LFM signal in the presence of AWGN is analyzed, where the SNR is set to be 3 dB. The WVD of the noisy LFM signal is provided in Figure 1a, and correspondingly, the contour of the computed WVD is presented in Figure 1b. From Figure 1, it can observe the TF peaks representing the noisy LFM signal, but there exist serious cross-terms in the TF plane where we expect no energy at all. The presence of the cross-terms of the analyzed signal in the TF plane does not possess any physical meaning, which makes proper signal interpretation very difficult; this is the main drawback of the WVD approach [30,31].

The WVD is a quadratic transformation, although it shows satisfactory TF aggregation property, the WVD of multi-component signal inevitably presents cross-term problem due to its bilinearity nature [30,31]. To deal with the cross-term problem, wavelet transform can be adopted, which is explained in the following section.

#### 2.2.2. Rearranged Wavelet Transform

CWT is a linear transform which does not suffer from the tough cross-term problem with the quadratic TF representation, and it can be expressed as [32]:

(13) $$W_{y}\left(a,b;\psi \right)=\int_{-\infty }^{+\infty }y_{a}\left(t\right)\psi_{a,b}^{*}\left(t\right)dt=\int_{-\infty }^{+\infty }y_{a}\left(t\right)\frac{1}{\sqrt{\left|a\right|}}\psi^{*}\left(\frac{t-b}{a}\right)dt$$

where $y_{a}\left(t\right)$ denotes the analytic form of the received GNSS signal; a is a scale parameter $\left(a\neq 0\right)$ representing the degree of compression; b denotes a translation parameter determining the location of the wavelet; the function $\psi \left(t\right)$ is called the mother wavelet, which is a continuous function both in time and frequency domains and usually used as a source function to produce daughter wavelets $\psi_{a,b}\left(t\right)=1/\sqrt{\left|a\right|}\psi^{*}\left(\frac{t-b}{a}\right)$, i.e., the translated and scaled version of the mother wavelet $\psi \left(t\right)$; and $\left(*\right)$ means the operation of conjugation. 

Since the integral of the squared amplitude of the CWT is proportional to the energy of the analyzed signal, the squared modulus of the CWT can be defined as scalogram, written as follows:

(14) $$SC_{y}\left(a,b;\psi \right)={\left|W_{y}\left(a,b;\psi \right)\right|}^{2}=\frac{1}{\left|a\right|}{\left|\int_{-\infty }^{+\infty }y_{a}\left(t\right)\psi^{*}\left(\frac{t-b}{a}\right)dt\right|}^{2}$$

where the scalogram is the signal energy distribution in the time-scale space, which can be used to represent the changing information of the analyzed signal in the TF plane; a is the stretching parameter that can adjust the daughter wavelet function’s oscillation frequency $f=f_{0}/a$, and $f_{0}$ is the center frequency of the mother wavelet $\psi \left(t\right)$.

In this paper, Morlet wavelet is chosen to extract linear frequency modulation feature of the sweep interfering signal. The Morlet wavelet kernel is given as [33,34]:

(15) $$\;\psi \left(t\right)=e^{-\frac{t^{2}}{2}}e^{j\omega_{0}t}$$

where t is the time, and $\omega_{0}$ denotes the angular frequency of the mother wavelet.

By substituting Equation (15) to Equation (13), the Morlet wavelet transform can be obtained, which is expressed as:

(16) $$T_{y}\left(t,a;\psi \right)=\int_{-\infty }^{+\infty }y_{a}\left(s\right)\frac{1}{\sqrt{\left|a\right|}}e^{-\frac{1}{2}{\left(\frac{s-t}{a}\right)}^{2}}e^{j\omega_{0}\left(\frac{t-s}{a}\right)}ds$$

where $y_{a}\left(s\right)$ is the input analytic signal, a represents the scale factor and t denotes the shift factor.

**Figure 2:**
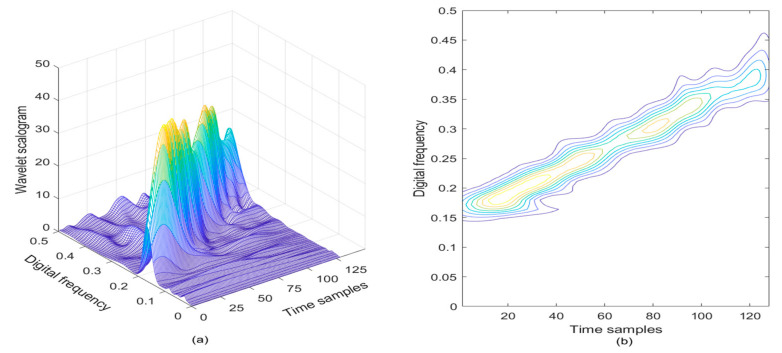
The wavelet scalogram of the LFM signal in the presence of AWGN, where the SNR is set to be 3 dB. (**a**) Wavelet scalogram; (**b**) Contour of wavelet scalogram.

In Figure 2a, the wavelet scalogram of the noisy LFM signal is provided, and correspondingly, the contour of the computed wavelet scalogram is presented in Figure 2b. From Figure 2, the TF energy peaks can be observed in the TF plane in the absence of cross-terms, which can almost represent the frequency modulation law of the LFM signal with the AWGN; but from Figure 2b, the wavelet scalogram shows very poor TF localization property in the TF plane, which shows unsatisfactory TF resolution in the LFM signal detection. 

Since the concept of rearrangement or reassignment can be used to improve the TF energy aggregation property in the signal detection applications [11,12], similarly, in this paper, to deal with the TF resolution problem with the wavelet scalogram, the rearrangement algorithm has been adopted to strengthen and concentrate the TF energy peaks in the signal detection. The TF resolution of the wavelet scalogram can be adjusted by changing the scale parameter a. At high frequencies, the scalogram provides a high time resolution but a low frequency resolution; while at low frequencies, the scalogram shows a high frequency resolution but a low time resolution. The TF resolution of the scalogram is bounded by the Heisenberg uncertainty principle. The scalogram $SC_{y}\left(t,a;\psi \right)$ represents the signal average energy density located in a certain local area geometrically centered in the point $\left(t,f\right)$ within the TF plane. The TF energy distribution in the local area is usually not geometrically symmetric, which will degrade the TF aggregation property of the energy distributions; therefore, it is not appropriate to assign the average energy density value to the geometric center of that considered local area [11,12]. 

One possible way to solve this problem is to reallocate the signal average energy density value to the gravity center $\left(\hat{t},\hat{f}\right)$ in that local domain, which more reasonably represents these TF energy distributions [11,12]. The rearrangement operation can be used to enhance the TF energy localization property, when the rearrangement is introduced to the wavelet scalogram, the energy distribution at any point $\left(t,f\right)$ in the TF plane will be moved to the corresponding new point $\left(\hat{t},\hat{f}\right)$, which is the gravity center of the signal energy distribution around the original point $\left(t,f\right)$. In this way, the rearranged wavelet scalogram obtained with the Morlet wavelet transform can be provided as:

(17) $$SC_{y}^{\left(r\right)}\left(t^{\prime },a^{\prime };\psi \right)=\int_{-\infty }^{+\infty }\int_{-\infty }^{+\infty }{\left(\frac{a^{\prime }}{a}\right)}^{2}SC_{y}\left(t,a;\psi \right)\delta \left(t^{\prime }-\hat{t}\left(y;t,a\right)\right)\delta \left(a^{\prime }-\hat{a}\left(y;t,a\right)\right)dtda$$

where $SC_{y}^{\left(r\right)}\left(t^{\prime },a^{\prime };\psi \right)$ is the rearranged wavelet scalogram; $\hat{t}\left(y;a,b\right)$ and $\hat{a}\left(y;a,b\right)$ are the corresponding reassigned values which determines the coordinates of the gravity center of the signal’s energy distribution; and $\delta \left(\cdot \right)$ denotes the Dirac delta function.

Furthermore, the rearrangement operator is given as:

(18) t^t,a;ψ=t−ReaTyt,a;τψTy*t,a;ψ|Tyt,a;ψ|2

(19) f^t,a;y=f0a^t,a;y=f0a+ImTyt,a;DψTy*t,a;ψ2πaTyt,a;ψ2

where $\tau_{\psi }$ and $D_{\psi }$ are the operations of multiplication and differentiation by the running variable, respectively, i.e., $\tau_{\psi }\left(t\right)=t\cdot \psi \left(t\right)$, $D_{\psi }\left(t\right)=\frac{d\psi \left(t\right)}{dt}$; $f_{0}$ is the center frequency of the mother wavelet $\psi \left(t\right)$; $T_{y}\left(t,a;\psi \right)$ is the Morlet wavelet transform of the analyzed signal $y_{a}\left(t\right)$. 

**Figure 3:**
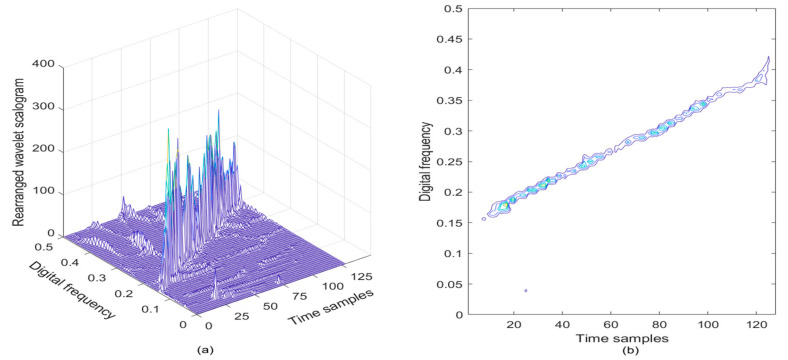
Rearranged wavelet scalogram of the LFM signal in the presence of AWGN, where the SNR is set to be 3 dB. (**a**) Rearranged wavelet scalogram; (**b**) Contour of rearranged wavelet scalogram.

In Figure 3a, the reassigned wavelet scalogram of the noisy LFM signal is provided, and correspondingly, the contour of the reassigned wavelet scalogram is given in Figure 3b. From Figure 3, it is easy to observe that the TF energy peaks are squeezed and concentrated in a linear region of the TF plane, presenting improved TF aggregation performance, thus the modulation law of the LFM signal can be roughly characterized. 

### 2.3. GNSS Interference Based on Rearranged Wavelet–Hough Transform

#### 2.3.1. Hough Transform

**Figure 4:**
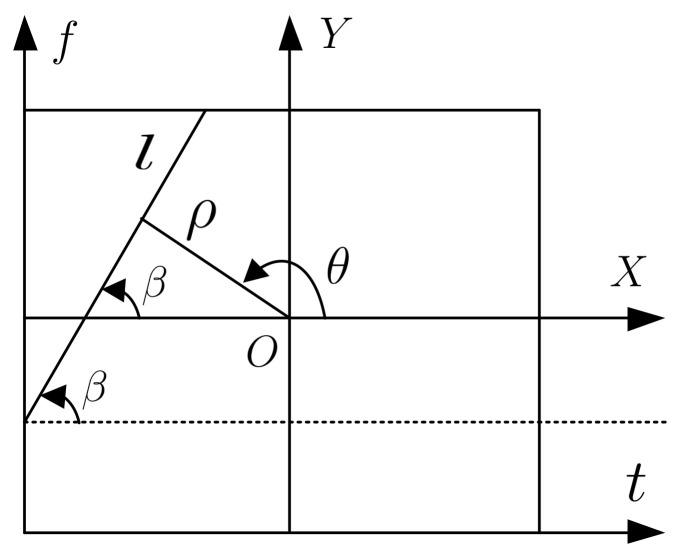
Scheme of Hough transform.

Hough transform can be used to detect the line of frequency modulation law for a given signal in the TF image space. The principle of Hough transform can be explained in Figure 4, where the center of a rectangular TF image is assumed to be the origin O of a Cartesian coordinate system XOY and the TF image size is set to be L×H. If the position of a pixel in the TF image is represented by $\left(t,f\right)$, then from Figure 4, it is easy to know that $x=t-\frac{L}{2}$, $y=f-\frac{H}{2}$. Concerning a straight line l in the TF space, the norm form of the Hough transform is given as follows [15]:

(20) xcosθ+ysinθ=ρ

where ρ is the distance from the origin to the closest point on the straight line l, θ denotes the angle between the X axis and the normal of the straight line l through the origin O. 

If a single point in the TF image is considered, then all the straight lines passing through this point definitely correspond to a unique sinusoidal curve in the ρ-θ plane. Therefore, it is easy to conclude that a set of two or more points which determines a straight line in the XOY system will produce corresponding sinusoidal curves which goes through a particular point $\left(\rho ,\theta \right)$ in the ρ-θ space which corresponds to the considered straight line. In this way, the problem of detecting collinear points in a line in the image space can be reasonably converted to the problem of searching for concurrent sinusoidal curves in the parameter space.

#### 2.3.2. Wigner–Hough Transform

When considering the detection of the LFM interfering signal (sweep interference) by using the WVD, if the undesired cross-term is not considered, the auto-term’s energy distribution is concentrated on a straight line representing the signal’s frequency modulation law in the TF plane [15]. If the Hough transform is combined with the WVD, the problem of detecting global straight line representing the LFM interference’s frequency modulation law in the TF plane can be converted to the problem of finding the corresponding energy peak focused on a particular point in the ρ-θ plane [15]. Therefore, the Wigner–Hough transform (WHT) can be formed, which is defined as [15,27]:

(21) $$WHT\left(f_{0},k\right)=\int_{-\infty }^{+\infty }\int_{-\infty }^{+\infty }y_{a}\left(t+\frac{\tau }{2}\right)y_{a}^{*}\left(t-\frac{\tau }{2}\right)e^{-j2\pi \left(f_{0}+kt\right)\tau }d\tau dt$$

where $y_{a}\left(t\right)$ is the analytical form of the input signal $y\left(t\right)$, $y_{a}^{*}\left(t\right)$ denotes the complex conjugate of $y_{a}\left(t\right)$, $f_{0}$ represents the initial frequency and k is the chirp rate. 

**Figure 5:**
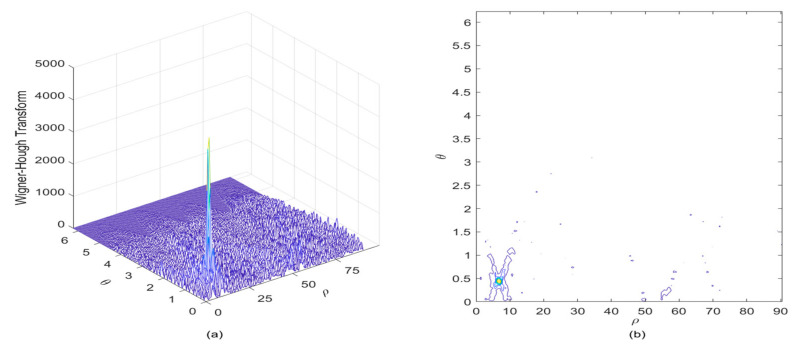
Wigner–Hough transform of the LFM signal in the presence of AWGN, where the SNR is set to be 3 dB. (**a**) Wigner–Hough transform; (**b**) Contour of Wigner–Hough transform.

In Figure 5, the WHT of the noisy LFM signal is presented in the ρ-θ plane, it is easy to observe that an energy peak denoting the LFM signal is concentrated in a limited point area, thus the LFM signal feature can be distinguished from that of the AWGN component. It can be known that the problem of detecting the straight line which represents the frequency modulation law of the LFM signal in the TF plane has been converted to the easily solved problem of searching for the energy peak position of the WHT in the ρ-θ space.

The characteristic parameters of the LFM signal can be further determined by using the Hough transform. In Figure 4, it can be assumed that there are $N_{f}$ sampling points on the frequency axis, then there exist $N_{t}$ sampling points on the time axis which satisfies $\tan\;\beta =-\cot\;\theta =N_{f}/N_{t}$. Therefore, the actual frequency modulation slope of the LFM interference is given as:

(22) $$k=\tan\beta \cdot \frac{\Delta f}{\Delta t}=\;-\cot\;\theta \cdot \;\frac{\Delta f}{\Delta t}$$

where β is the angle between the straight line l and the t axis, $\beta =\theta -\frac{\pi }{2}$; Δf is the frequency resolution, which is equal to $\frac{f_{s}}{2L}$; Δt means the time resolution, which is equal to $\frac{1}{f_{s}}$; and $f_{s}$ denotes the sampling rate.

According to the geometric relationship shown in Figure 4, it is easy to obtain the relationship between the initial frequency $f_{0}$ and the polar coordinates $\left(\rho ,\theta \right)$ of the energy peak in the parameter space, shown as:

(23) $$f_{0}=\left(\frac{H}{2}+\frac{L}{2}\cot\theta +\frac{\rho }{\sin\theta }\right)\Delta f$$

When the WHT is used in sweep interference detection for GNSS applications, the colinear points $\left(t_{i},f_{i}\right)$ (i=0,⋯,N−1, and N is the number of points in the straight line representing the frequency modulation law of the analyzed signal, which is equal to the number of input signal samples) in the frequency modulation line within the TF image are mapped into a particular point $\left(\rho ,\theta \right)$ with the polar form whose position is determined by the energy peak of the WHT which is formed by N concurrent sinusoidal curves in the ρ-θ parameter space; in this way the characteristic parameters such as chirp rate k and initial frequency $f_{0}$ of the LFM signal can be further estimated.

#### 2.3.3. Rearranged Wavelet–Hough Transform

The observed straight line denoting the frequency modulation law of the sweep interfering signal performed by using rearranged wavelet transform is usually described with the Cartesian coordinates $\left(x,y\right)$ (equivalent to $\left(t,f\right)$) in the image space, and by using Hough transform it can be converted into the polar coordinates $\left(\rho ,\theta \right)$ in the parametric space, which can be written as:

(24) $$\rho =\sqrt{x^{2}+y^{2}}\sin\left(\theta +\arctan\frac{x}{y}\right)$$

where ρ is the normal distance between the straight line and the origin O of the XOY coordinate system, and θ denotes the angle between the normal and the X axis, $\theta \in \left[0,\pi \right]$. 

From Cartesian coordinates to polar coordinates, the point-line duality has been converted to the point-sinusoidal curve duality through Hough transform; in this way, the detection of linear lines in the TF image space can be converted to detect intersection points of sinusoidal curves in the $\left(\rho ,\theta \right)$ parameter space. Therefore, to improve GNSS interference detection performance, the rearranged wavelet scalogram can be combined with the Hough transform to develop a novel RWHT, which can be determined as:

(25) $$RWHT_{y}\left(f,k\right)=\int_{-\infty }^{+\infty }\int_{-\infty }^{+\infty }SC_{y}^{\left(r\right)}\left(t,\upsilon \right)\delta \left(\upsilon -f-kt\right)dtd\upsilon$$

where $SC_{y}^{\left(r\right)}$ is the reassigned wavelet scalogram in the TF domain, $\delta \left(\cdot \right)$ denotes the Dirac delta function.

Equation (25) can be also rewritten in the form of polar coordinates [15], provided as:

(26) $$RWHT_{y}\left(\rho ,\theta \right)=\int_{-\infty }^{+\infty }SC_{y}^{\left(r\right)}\left(t,\frac{1}{\sin\theta }\left(\rho -t\;\cdot \cos\theta \right)\right)dt$$

It can be seen from Equation (26) that through Hough transform, the original straight line denoting the sweep interring signal’s frequency modulation law which is represented by the rearranged wavelet scalogram in the TF plane can be mapped to the concurrent sinusoidal curves which are concentrated in a particular point in the form of polar coordinates within the $\left(\rho ,\theta \right)$ parameter plane, this is the core idea of the proposed RWHT algorithm for GNSS interference detection applications. 

When dealing with the sweep interference present in the received GNSS signal, since the concurrent sinusoidal curves which represent the collinear points on the sweep interference frequency modulation line are intersected in the same point within the $\left(\rho ,\theta \right)$ parameter space, thus an energy peak will be formed in this point domain where the RWHT energy distributions are greatly strengthened and concentrated. This particular RWHT energy distribution feathers can be used to differentiate the sweep interference from the AWGN and useful GNSS signal components in the parameter space. By searching for the RWHT energy peak position, the characteristic paraments such as initial frequency and chirp rate of the sweep interference can be effectively estimated. 

**Figure 6:**
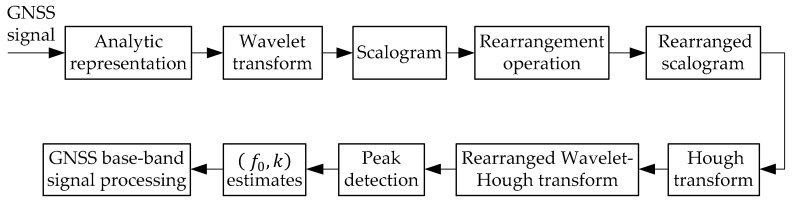
Interference detection by using the rearranged wavelet–Hough transform for GNSS receivers.

The proposed GNSS interference detection method based on RWHT is clearly explained in Figure 6. The received GNSS signal in the analytic form is wavelet-transformed, then the scalogram is calculated, which is to be redistributed by using the rearrangement to obtain rearranged scalogram. The rearranged scalogram presents improved TF aggregation property, but in low jammer-to-noise ratio (JNR )condition of GNSS interference detection, the TF energy distribution discontinuous phenomenon may occur in the TF plane; therefore, to deal with such discontinuity problem with the rearranged scalogram, the Hough transform is introduced and combined with the rearranged scalogram, theoretically, thus the RWHT can be obtained. All the points in the straight line represented by the rearranged scalogram in the TF image space will correspond to the RWHT energy peak concentrated in a determined point $\left(\rho ,\theta \right)$ within the parameter space. 

When the RWHT is used in the detection of sweep interference present in the received GNSS signal, a sharp energy peak definitely appears in a specific point domain within the parameter space, presenting all the information of the frequency modulation law of the linear chirp interference. By searching for the local RWHT peak and determining this particular point position within the parameter space where the RWHT peak energy concentrates, the sweep interfering signal can be effectively detected, and furthermore the initial frequency and chirp rate of the linear chirp interference present in the received GNSS signal can be precisely estimated by the peak detection process.

## 3. Results

**Figure 7:**
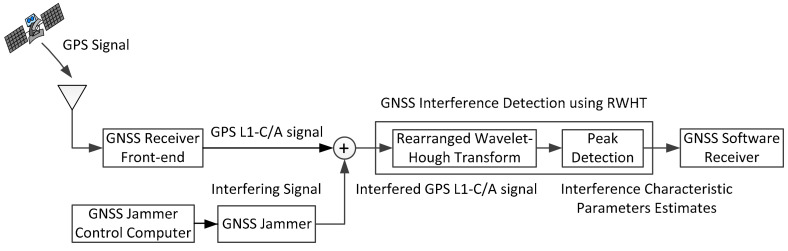
The scheme of the GNSS interference detection test in the lab.

**Figure 8:**
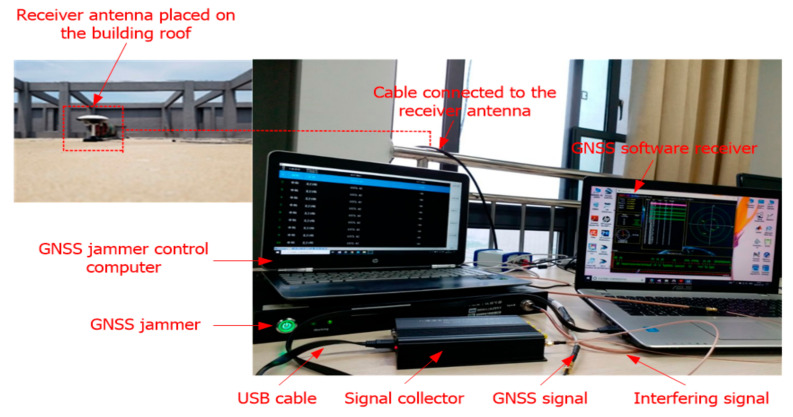
The experimental setup of the GNSS interference detection test.

In this section, the performance of GNSS interference detection based on rearranged wavelet–Hough transform was analyzed in comparison with the traditional interference detection techniques such as WVD and WHT. The scheme of the GNSS interference detection test is illustrated in Figure 7, and the experimental setup for collecting the GPS-L1 C/A signal corrupted by interference is depicted in Figure 8. A jammer controlled by a computer was used to produce interference, which was added to the GPS L1-C/A samples collected by the GNSS software receiver, whose receiver front end was connected to the receiver antenna placed on the building roof through a cable. The combined signal was captured using a GNSS signal collector and sent to the GNSS software receiver implemented on another computer through a USB cable.

To demonstrate the validity and effectiveness of the proposed RWHT-based interference detection method for GNSS receivers, several tests were performed, which were characterized by the corresponding parameters provided in Table 1. In the experiment, the carrier-to-noise power density ratio $C/N_{0}$ of the received GPS L1-C/A signal was 40 dB-Hz, the intermediate frequency $f_{IF}$ was set to 39.96 MHz and the sampling frequency $f_{s}$ was set to 20.47 MHz in compliance with the band-pass sampling theorem. The collected GPS L1-C/A signal in zero-mean AWGN was jammed by a constant amplitude LFM interference (i.e., linear chirp) or CW interference, which were chosen as the typical test bench in GNSS interference detection. When considering the linear chirp interference present in the received GPS L1-C/A signal, the initial frequency $f_{0}$ of the linear chirp interference was set to 4.9 MHz, the sweep period $t_{j}$ of the linear chirp interference was set to 0.1 ms and the chirp rate k was chosen to be −1.0 × 10^4^ MHz/s.

**Figure 9:**
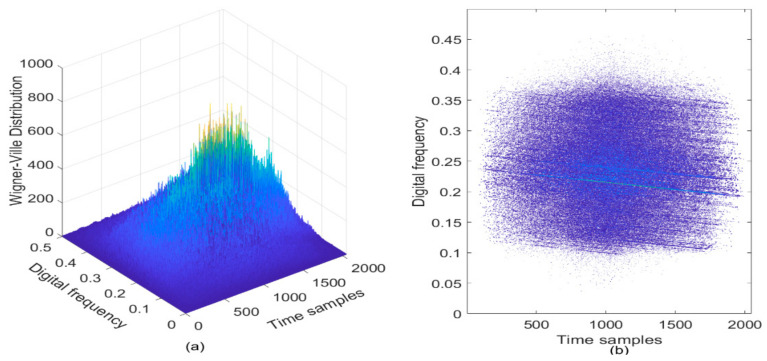
The WVD of the GPS L1-C/A signal in the presence of sweep interference. $C/N_{0}\;$ = 40 dB-Hz, JNR = −2 dB. (**a**) WVD; (**b**) Contour of WVD.

In Figure 9a, the WVD of the GPS L1-C/A signal in the presence of sweep interference is depicted, and the contour of the WVD is correspondingly shown in Figure 9b. Although a straight line denoting the frequency modulation law of the sweep interference can be seen in the TF plane, serious cross-terms can be also observed due to the bilinear nature of the WVD, which inevitably cause error to the estimation of the instantaneous frequency of the sweep interfering signal and bring difficulties in the detection of interference, thus, the GNSS interference characteristic parameters cannot be correctly extracted using the WVD.

**Figure 10:**
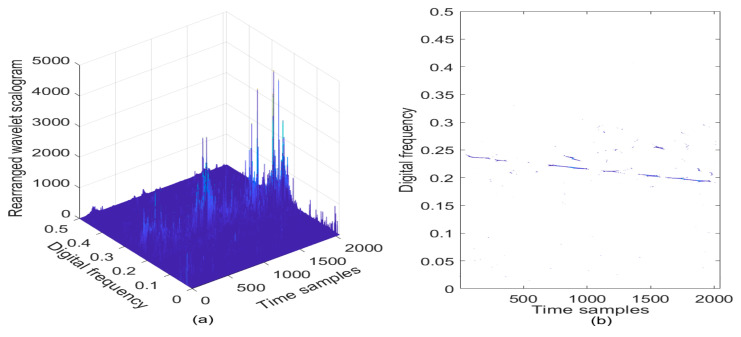
Rearranged wavelet scalogram of the GPS L1-C/A signal in the presence of sweep interference. $C/N_{0}\;$ = 40 dB-Hz, JNR = −2 dB. (**a**) Rearranged wavelet scalogram; (**b**) Contour of rearranged wavelet scalogram.

To mitigate the cross-term interference with the WVD, the rearranged wavelet scalogram of the interfered GPS L1-C/A signal is shown Figure 10. It can be observed that the cross-term is effectively suppressed in the TF plane and the TF energy peaks are linearly distributed on the straight line denoting the frequency modulation law of the sweep interference present in the received GPS L1-C/A signal. It is easy to know that the interference detection ability with the rearranged wavelet transform outperforms the WVD, as verified in Figure 10. However, the discontinuity phenomena of the TF energy distribution along the straight line of frequency modulation law of the sweep interference occurs in the TF plane due to use of rearrangement operation in the rearranged wavelet transform, which possibly causes error to the instantaneous frequency estimation of interfering signal.

**Figure 11:**
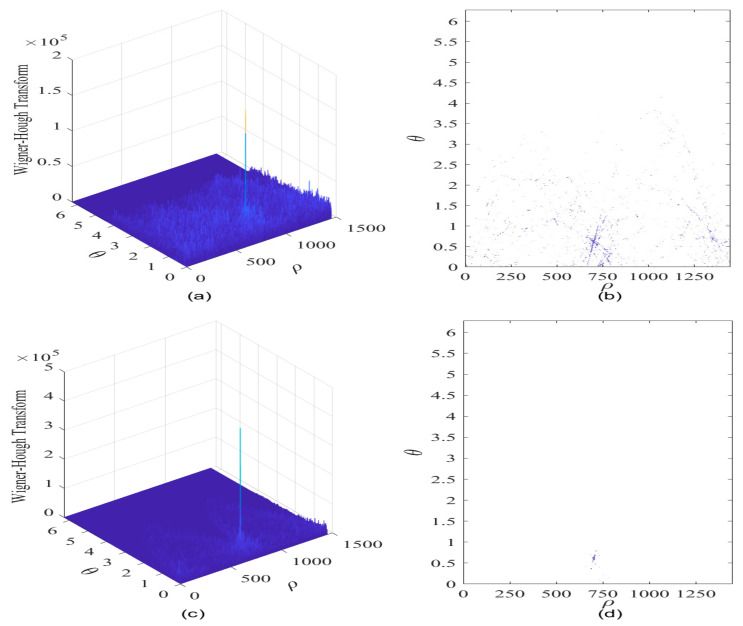
The Wigner–Hough transform of the GPS L1-C/A signal in the presence of sweep interference. $C/N_{0}\;$ = 40 dB-Hz. (**a**) WHT, JNR = −8 dB; (**b**) Contour of WHT; (**c**) WHT, JNR = −2 dB; (**d**) Contour of WHT.

The Hough transform can be used to mitigate the cross-terms present in the TF energy distributions. In Figure 11a, the WHT of the interfered GNSS signal is shown in case of JNR equal to −8 dB; and correspondingly, the contour of the WHT is given in Figure 11b. From Figure 11a, it can be found that a WHT peak denoting the sweep interference occurs in the ρ-θ plane, but the energy peak position is relatively fuzzy in the parameter space, thus it is difficult to determine the peak position accurately due to the influence of the existence of undesired cross-terms, as verified in Figure 11b. It can be known that errors can be possibly brought into the estimation of characteristic parameters of the sweep interfering signal in the low JNR condition. 

When the JNR of the sweep interference is set to be −2 dB, the WHT of the interfered GNSS signal is presented in Figure 11c and the corresponding contour of the WHT is given in Figure 11d. It can be observed that concurrent sinusoidal curves representing the collinear points in the straight line of frequency modulation law of the sweep interference are intersected in a specific point area within the ρ-θ plane, where the energy distributions are accumulated within such point domain in the parameter space, therefore, when the energy peak position of the WHT is roughly determined in the parameter space, the GNSS interference feature can be possibly extracted. 

**Figure 12:**
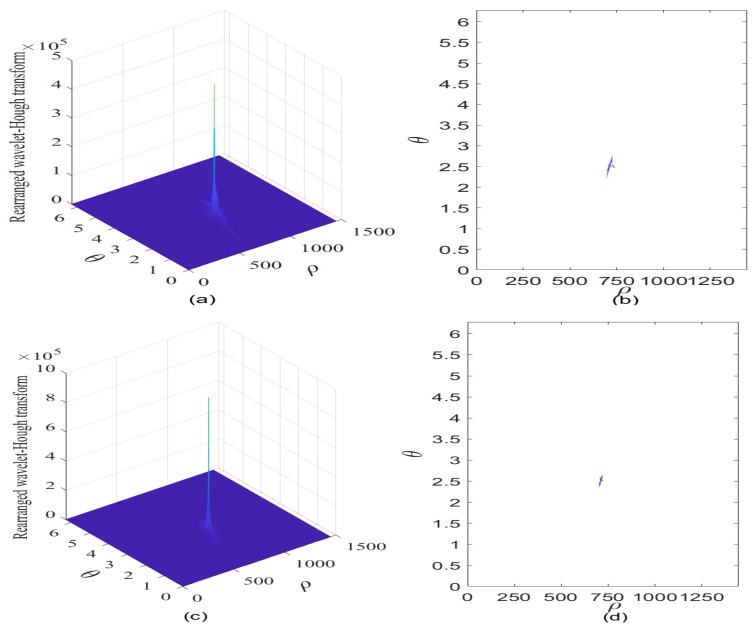
The rearranged Wavelet–Hough transform of the GPS L1-C/A signal in the presence of sweep interference. $C/N_{0}\;$ = 40 dB-Hz. (**a**) Rearranged Wavelet–Hough transform, JNR = −8 dB; (**b**) Contour of rearranged Wavelet–Hough transform; (**c**) Rearranged Wavelet–Hough transform, JNR = −2 dB; (**d**) Contour of rearranged Wavelet–Hough transform.

To further improve the interference detection performance for GNSS receivers, the RWHT of the interfered GPS L1-C/A signal is provided in Figure 12a in case of JNR equal to −2 dB; and its corresponding contour is given in Figure 12b. It can be seen that a distinct peak occurs in the parameter space, and the intensity of such RWHT energy distributions representing the contribution of sweep interference present in the received GPS L1-C/A signal is accumulated and strengthened in a particular point area where the concurrent sinusoidal curves are intersected forming a strong RWHT energy peak, denoting the collinear points distributed on the straight line of reassigned wavelet scalogram of the interfered GPS L1-C/A signal in the TF plane; in this way, the discontinuity problem of the rearranged wavelet transform in the TF space can be effectively solved when adopting the proposed RWHT-based interference detection method in the ρ-θ plane. Moreover, in comparison to the WHT result shown in Figure 11b, the RWHT peak position can be determined in the ρ-θ plane even in case of JNR equal to −8 dB, it is easy to know that improved interference detection ability can be obtained with the developed RWHT technique, as verified in Figure 12b.

In case of JNR equal to −2 dB, the RWHT of the interfered GNSS signal is provided in Figure 12c, and its contour is shown in Figure 12d. It is clear to observe that a very sharp peak occurs in a point domain within the ρ-θ space, where the RWHT energy distributions much concentrate in such intersection point domain. Meanwhile, in the other area excluding the intersection point domain of the concurrent sinusoidal curves existed in the ρ-θ plane, the RWHT energy distributions contributed from the white Gaussian noise and the useful GPS L1-C/A signal components are reduced significantly compared with the WHT energy peak level located in the intersected point domain, and the RWHT energy distributions of these signal components within such area in the ρ-θ plane become much sparser in comparison to the WHT case; moreover, the cross-terms with the RWHT are effectively suppressed in comparison to the WHT approach. Therefore, the sweep interfering signal can be clearly distinguished from the other terms of white Gaussian noise and useful GPS L1-C/A signal and its characteristic features can be easily extracted. Furthermore, in comparison to the case of JNR equal to −8 dB, the dimension of the RWHT energy peak point domain where the concurrent sinusoidal curves are intersected is much reduced, which means that much improved estimation precision for the sweep interference characteristic parameters can be obtained since more accurate determination of the RWHT energy peak position in the ρ-θ space can be made. The initial frequency of the sweep interfering signal is 4.9207 MHz, and the estimated chirp rate of the sweep interfering signal is −1.0051 × 10^4^ MHz/s.

**Figure 13:**
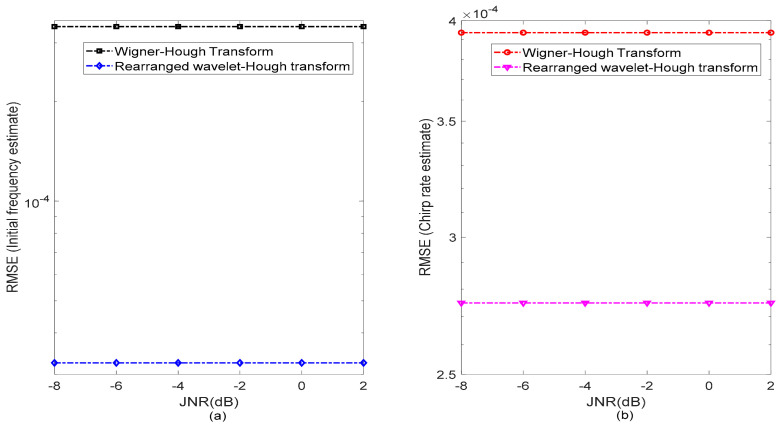
The RMSE of the estimated chirp rate and initial frequency of the sweep interference present in the received GPS L1-C/A signal by using Wigner–Hough transform and rearranged Wavelet–Hough transform, respectively. $C/N_{0}$ = 40 dB-Hz. (**a**) RMSE of the estimated initial frequency of the sweep interference; (**b**) RMSE of the estimated chirp rate of the sweep interference.

To comprehensively evaluate the GNSS detection performance of the proposed RWHT method, root mean square error (RMSE) has been used in the analysis for the estimates of the initial frequency and chirp rate of the sweep interference in comparison to the WHT approach. In different JNR scenarios, the RMSE results for the estimated initial frequency and chirp rate values of the sweep interference present in the received GPS L1-C/A signal are provided in Figure 13. It can be found that the RMSE results of the RWHT and WHT remain almost unchanged with changes of the JNR values of the sweep interfering signal, indicating that these two methods present stability and robustness in the interference detection and feather parameters estimation. 

In details, in Figure 13a, the RMSE level of the GNSS sweep interference initial frequency estimate with the WHT is almost kept at $3.3\times 10^{-4}$ level, while the RMSE level of the GNSS sweep interference initial frequency estimate with the RWHT is kept at about $3.2\times 10^{-5}$ level, presenting about 90.3% initial frequency estimation precision improvement over the WHT approach. Similarly, in Figure 13b, the RMSE level of the chirp rate estimate obtained with the WHT nearly remains at the level of $3.9\times 10^{-4}$, while the RMSE of the estimated chirp rate achieved with the RWHT almost keeps at the level of $2.7\times 10^{-4}$, it can be known that the proposed RWHT method has a 30.8% chirp rate estimation precision enhancement over the WHT approach. From the RMSE analysis results, it is easy to know that regardless of the estimation of the initial frequency or the chirp rate of the sweep interference present in the received GPS L1-C/A signal, the RWHT technique shows much improved interference detection performance for GNSS receivers. 

**Figure 14:**
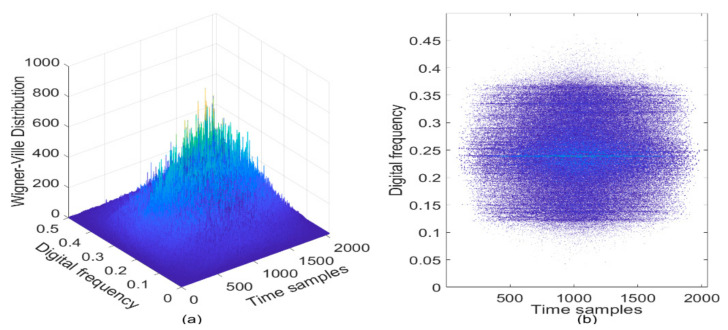
The WVD of the GPS L1-C/A signal in the presence of continuous wave interference. $C/N_{0}$ = 40 dB-Hz, JNR = −2 dB. (**a**) WVD; (**b**) Contour of WVD.

Moreover, the proposed RWHT method is expected to be also valid in the detection of CW interference since it can be considered to be a special case of LFM interfering signal with a fixed carrier center frequency [13]. In the experiment, the GNSS CW interference characterized by J/N = −2 dB is considered in the disturbing scenario. In Figure 14a, the WVD of the GPS L1-C/A signal in the presence of CW interference is provided, and correspondingly, the contour of the obtained WVD is given in Figure 14b. From Figure 14, a horizontal straight line can be faintly observed in the TF plane with a fixed frequency, which denotes the TF characteristic of the CW interference; but there also exist very severe cross-terms in the TF plane at the same time, which will bring serious difficulties to the correct understanding of the CW interference present in the received GPS L1-C/A signal.

**Figure 15:**
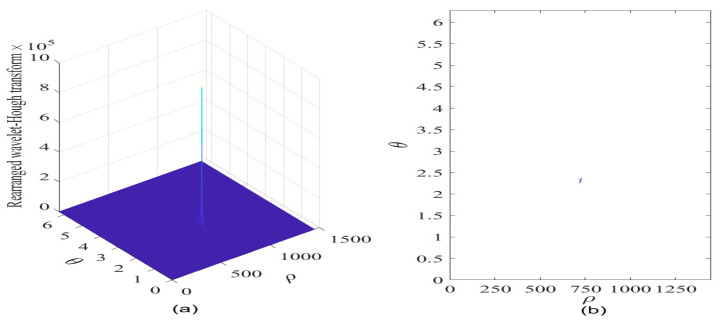
The rearranged Wavelet–Hough transform of the GPS L1-C/A signal in the presence of continuous wave interference. $C/N_{0}$ = 40 dB-Hz, JNR = −2 dB. (**a**) Rearranged Wavelet–Hough transform; (**b**) Contour of rearranged Wavelet–Hough transform.

In Figure 15a, the proposed RWHT of the GPS-L1 C/A signal in the presence of CW interference is evaluated, and its contour is accordingly shown in Figure 15b. From Figure 15, a very sharp vertical peak of the RWHT energy distribution can be observed in a particular point domain within the ρ-θ parameter space, and the RWHT energy highly concentrates in this point, whose position can be used to estimate the characteristic parameters of the CW interference; in the other area except the point domain within the ρ-θ parameter space, the RWHT energy distributions corresponding to the white Gaussian noise and the useful GPS L1-C/A signal components are effectively suppressed to a negligible level when compared with the RWHT energy peak. The proposed RWHT method completely removes the cross-term artifacts within the bilinear TF distributions such as WVD, and at the same time it presents satisfactory energy aggregation property in GNSS interference detection.

In summary, it is known that the proposed RWHT method is not only effective for sweep interference detection, but also suitable for CW interference detection for GNSS receivers.

## 4. Discussion

From the GNSS sweep interference detection results, the proposed RWHT method can be used to detect the GNSS sweep (linear chirp) interference effectively since it successfully deals with the cross-term problem and presents much improved energy localization property. The estimation of the characteristic parameters such as the initial frequency and chirp rate of the sweep interfering signal has been performed, and the corresponding quantitative metric RMSE results have been compared between the proposed RWHT method and the WHT approach. The RWHT method shows much improved precision in the estimation of the characteristic parameters of the sweep interference present in the received GPS-L1 C/A signal. 

Furthermore, in this paper, this proposed RWHT method has been experimentally verified to be valid and effective in the detection of CW interference for GNSS receivers. The RMSE analysis of this proposed RWHT method in dealing with the CW interference for GNSS applications will be evaluated in the future research works.

In addition, since both Beidou and GPS signals use spread spectrum communication and code division multiple access (CDMA) technologies [1], the proposed RWHT method is expected to be effective in the detection of interference present in the Beidou signals. The rationality behind this proposed RWHT method is based on the huge difference of the energy distributions contributed from the useful GNSS signal and the interfering signal (including sweep interference and CW interference) when they are evaluated in the ρ-θ parameter space, respectively. This will be further analyzed in our future research works. 

## 5. Conclusions

In this paper, a novel GNSS interference detection method based on rearranged wavelet–Hough transform has been proposed. To prove the validity and effectiveness of the developed technique, a comprehensive interference detection performance evaluation has been performed in comparison to the existed TF analysis approaches such as WVD, WHT, and rearranged wavelet scalogram. The interference detection tests have been performed on the real GPS L1-C/A signal in the presence of sweep interference or CW interference to verify the theoretical analyses.

From the experimental results, the traditional WVD approach shows unsatisfactory interference detection performance since it is suffered from the serious cross-term problem; the rearranged scalogram can partially suppress cross-terms, but it presents TF energy distribution discontinuity problem in the TF plane due to the rearrangement operation, which degrades the GNSS interference detection performance; when the WVD is combined with the Hough transform, the obtained WHT can partially alleviate the cross-terms present in the TF plane; when the Hough transform is combined with the rearranged scalogram, it is of vital importance that the developed RWHT method effectively suppresses the undesired cross-terms since the RWHT energy distributions are greatly strengthened and mainly concentrated in a particular limited point domain within the parameter space, and at the same time, the RWHT method presents satisfactory energy aggregation property since the rearrangement operation is made, this can be considered to be the main novelty of this paper. It is clear to know that the proposed RWHT method provides significant performance improvement in GNSS interference detection when compared with the existed TF analysis approaches.

The proposed interference detection method by using RWHT has been proven to be very effective to remove the cross-terms within the traditional bilinear TF distributions and improve the TF energy aggregation property at the same time, which is critically important to the GNSS interference detection performance enhancement. Based on these achieved technical improvements, the RWHT-based GNSS interference detection method provides much improved performance over the WVD, rearranged wavelet scalogram, and WHT techniques.

In summary, by using the developed efficient RWHT algorithm, the proposed GNSS interference detection method is not only effective in GNSS sweep interference detection, but also valid in dealing with GNSS CW interference, which is very promising to be used in anti-interference design for GNSS receivers particularly working in the difficult and challenging interfering environments.

## Figures and Tables

**Table 1:** The experimental setting parameters in the GNSS interference detection.

Parameter	Value
Carrier-to-noise power density ratio, $C/N_{0}$	40 dB-Hz
Sampling frequency, $f_{s}$	20.47 MHz
Intermediate frequency, $f_{IF}$	39.96 MHz
Sweep interference initial frequency, $f_{0}$	4.9 MHz
Sweep interference sweep period, $t_{j}$	0.1 ms
Sweep interference chirp rate, k	−1.0 × 10^4^ MHz/s

## Data Availability

Not applicable.

## References

[1] Kaplan E.D. (2017). Understanding GPS/GNSS: Principles and Applications.

[2] Borio D., Dovis F., Kuusniemi H., Presti L.L. (2016). Impact and Detection of GNSS Jammers on Consumer Grade Satellite Navigation Receivers. Proc. IEEE.

[3] Borio D., O’Driscoll C., Fortuny J. Jammer impact on Galileo and GPS receivers. Proceedings of the 2013 International Conference on Localization and GNSS (ICL-GNSS).

[4] Ioannides R.T., Pany T., Gibbons G. (2016). Known vulnerabilities of Global Navigation Satellite Systems, status, and potential mitigation techniques. Proc. IEEE.

[5] Mitch R.H., Dougherty R.C., Psiaki M.L., Powell S.P., O’Hanlon B.W., Bhatti J.A., Humphreys T.E. Signal characteristics of civil GPS jammers. Proceedings of the 24th International Technical Meeting of The Satellite Division of the Institute of Navigation (ION GNSS 2011).

[6] Kraus T., Bauernfeind R., Eissfeller B. Survey of In-Car Jammers—Analysis and Modeling of the RF Signals and IF Samples (Suitable for Active Signal Cancelation). Proceedings of the 24th International Technical Meeting of The Satellite Division of the Institute of Navigation (ION GNSS 2011).

[7] Borio D., Camoriano L., Savasta S., Presti L.L. (2008). Time-frequency excision for GNSS applications. IEEE Syst. J..

[8] Balaei A.T., Dempster A.G. (2009). A statistical inference technique for GPS interference detection. IEEE Trans. Aerosp. Electron. Syst..

[9] Motella B., Savasta S., Margaria D., Dovis F. (2011). Method for assessing the interference impact on GNSS receivers. IEEE Trans. Aerosp. Electron. Syst..

[10] Sun K., Liu W., Xu H., Yang D. Interference Detection Based on Time-Frequency Analysis for GNSS Receivers. Proceedings of the 26th International Technical Meeting of the Satellite Division of the Institute of Navigation.

[11] Sun K., Jin T., Yang D. (2015). An improved time-frequency analysis method in interference detection for GNSS receivers. Sensors.

[12] Sun K., Jin T., Yang D. (2015). A new reassigned spectrogram method in interference detection for GNSS receivers. Sensors.

[13] Sun K., Zhang M., Yang D. (2016). A New Interference Detection Method Based on Joint Hybrid Time-Frequency Distribution for GNSS Receivers. IEEE Trans. Veh. Technol..

[14] Wang P., Cetin E., Dempster A.G., Wang Y., Wu S. (2017). GNSS interference detection using statistical analysis in the time-frequency domain. IEEE Trans. Aerosp. Electron. Syst..

[15] Sun K., Guo J. A Novel Interference Detection Method Based on Wigner-Hough Transform for GNSS Receivers. Proceedings of the 32nd International Technical Meeting of the Satellite Division of The Institute of Navigation (ION GNSS+ 2019).

[16] Lv Q., Qin H. (2019). A Joint Method Based on Time-Frequency Distribution to Detect Time-Varying Interferences for GNSS Receivers with a Single Antenna. Sensors.

[17] Amoroso F. (1983). Adaptive A/D converter to suppress CW interference in DSPN spread-spectrum communications. IEEE Trans. Commun..

[18] Amoroso F., Bricker J.L. (1986). Performance of the adaptive A/D converter in combined CW and Gaussian interference. IEEE Trans. Commun..

[19] Bastide F., Akos D., Macabiau C., Roturier B. Automatic gain control (AGC) as an interference assessment tool. Proceedings of the 16th International Technical Meeting of the Satellite Division of The Institute of Navigation (ION GPS/GNSS 2003).

[20] Gao G.X., De Lorenzo D.S., Walter T., Enge P. Acquisition and tracking of GIOVE-a broadcast L1/E5/E6 signals and analysis of DME/TACAN interference on receiver design. Proceedings of the European Navigation Conference on Global Navigation Satellite Systems (ENC-GNSS 2007).

[21] Palestini C., Pedone R., Villanti M., Corazza G.E. (2008). Integrated NAV-COM systems: Assisted code acquisition and interference mitigation. IEEE Syst. J..

[22] Kuriger G., Grant H., Cartwright A., Heirman D. (2003). Investigation of spurious emissions from cellular phones and the possible effect on aircraft navigation equipment. IEEE Trans. Electromagn. Compat..

[23] Raimondi M., Julien O., Macabiau C., Bastide F. Mitigating pulsed interference using frequency domain adaptive filtering. Proceedings of the 19th International Technical Meeting of the Satellite Division of The Institute of Navigation (ION GNSS 2006).

[24] Tani A., Fantacci R. (2008). Performance evaluation of a precorrelation interference detection algorithm for the GNSS based on nonparametrical spectral estimation. IEEE Syst. J..

[25] Hough P.V.C. (1962). Method and Means for Recognizing Complex Patterns. U.S. Patent.

[26] Ballard D.H. (1981). Generalizing the Hough Transform to Detect Arbitrary Shapes. Pattern Recognit..

[27] Barbarossa S. (1995). Analysis of multicomponent LFM signals by a combined Wigner-Hough transform. IEEE Trans. Signal Process..

[28] Auger F., Flandrin P. (1995). Improving the Readability of Time-Frequency and Time-Scale Representations by the Reassignment Method. IEEE Trans. Signal Process..

[29] Richard C., Lengelle R. (1997). Joint Recursive Implementation of Time-Frequency Representations and Their Modified Version by the Reassignment Method. Signal Process..

[30] Cohen L. (1994). Time-Frequency Analysis: Theory and Applications.

[31] Gröchenig K. (2001). Foundations of Time-Frequency Analysis.

[32] Lokenath D., Firdous A.S. (2017). Lecture Notes on Wavelet Transforms.

[33] Morlet J., Arens G., Fourgeau E., Giard D. (1982). Wave propagation and sampling theory, Part I: Complex signal land scattering in multilayer media. Geophysics.

[34] Morlet J., Arens G., Fourgeau E., Giard D. (1982). Wave propagation and sampling theory, Part II: Sampling theory and complex waves. Geophysics.

